# Paving the Way Toward Distinguishing Fallers From Non-fallers in Bilateral Vestibulopathy: A Wide Pilot Observation

**DOI:** 10.3389/fneur.2021.611648

**Published:** 2021-03-01

**Authors:** Nolan Herssens, Bieke Dobbels, Julie Moyaert, Raymond Van de Berg, Wim Saeys, Ann Hallemans, Luc Vereeck, Vincent Van Rompaey

**Affiliations:** ^1^Department of Rehabilitation Sciences, Ghent University, Ghent, Belgium; ^2^Department of Rehabilitation Sciences and Physiotherapy/Movant, Faculty of Medicine and Health Sciences, University of Antwerp, Antwerp, Belgium; ^3^Department of Otorhinolaryngology and Head & Neck Surgery, Antwerp University Hospital, Edegem, Belgium; ^4^Faculty of Medicine and Health Sciences, University of Antwerp, Antwerp, Belgium; ^5^Division of Balance Disorders, Department of Otorhinolaryngology and Head and Neck Surgery, Faculty of Health Medicine and Life Sciences, School for Mental Health and Neuroscience, Maastricht University Medical Centre, Maastricht, Netherlands; ^6^Faculty of Physics, Tomsk State University, Tomsk, Russia; ^7^RevArte Rehabilitation Hospital, Edegem, Belgium; ^8^Multidisciplinary Motor Centre Antwerp (M^2^OCEAN), University of Antwerp, Antwerp, Belgium

**Keywords:** bilateral vestibulopathy, falls, balance, gait, self-perceived disability, vestibular function

## Abstract

Patients with bilateral vestibulopathy (BVP) present with unsteadiness during standing and walking, limiting their activities of daily life and, more importantly, resulting in an increased risk of falling. In BVP patients, falls are considered as one of the major complications, with patients having a 31-fold increased risk of falling compared to healthy subjects. Thus, highlighting objective measures that can easily and accurately assess the risk of falling in BVP patients is an important step in reducing the incidence of falls and the accompanying burdens. Therefore, this study investigated the interrelations between demographic characteristics, vestibular function, questionnaires on self-perceived handicap and balance confidence, clinical balance measures, gait variables, and fall status in 27 BVP patients. Based on the history of falls in the preceding 12 months, the patients were subdivided in a “faller” or “non-faller” group. Results on the different outcome measures were compared between the “faller” and “non-faller” subgroups using Pearson's chi-square test in the case of categorical data; for continuous data, Mann–Whitney *U* test was used. Performances on the clinical balance measures were comparable between fallers and non-fallers, indicating that, independent from fall status, the BVP patients present with an increased risk of falling. However, fallers tended to report a worse self-perceived handicap and confidence during performing activities of daily life. Spatiotemporal parameters of gait did not differ between fallers and non-fallers during walking at slow, preferred, or fast walking speed. These results may thus imply that, when aiming to distinguish fallers from non-fallers, the BVP patients' beliefs concerning their capabilities may be more important than the moderately or severely affected physical performance within a clinical setting. Outcome measures addressing the self-efficacy and fear of falling in BVP patients should therefore be incorporated in future research to investigate whether these are indeed able to distinguish fallers form non-fallers. Additionally, information regarding physical activity could provide valuable insights on the contextual information influencing behavior and falls in BVP.

## Introduction

Bilateral vestibulopathy (BVP) is a chronic condition, characterized by a severely impaired or a bilateral loss of peripheral vestibular function (1, 2). In 2017, the diagnostic criteria for BVP were defined by the Bárány Society (3). These diagnostic criteria involve symptoms of oscillopsia during walking or quick head movements and unsteadiness during standing and walking (3). While the clinical presentation of the BVP population however remains very heterogenous [see (4) for a review], oscillopsia and unsteadiness are indeed reported by the majority of BVP patients. Both symptoms of oscillopsia and unsteadiness may result in BVP patients limiting their activities of daily life, making them more sedentary and move more rigidly to limit their symptoms which, ultimately, may result in an increased risk of falling (5, 6).

Falls are considered to be one of the major complications in BVP, with BVP patients having a 31-fold increased risk of falling compared to their healthy counterparts (7). Additionally, when compared to patients with other peripheral vestibular syndromes, patients with BVP were also found to have the highest risk of falls (8). Next to potential injuries and loss of independence related to a fall episode, secondary complications related to the fall, such as developing a concern for future falls, in turn, may result in social isolation and an important decline of the quality of life (9). Therefore, it would be beneficial to be able to highlight objective measures that could assess the BVP patients' fall risk more easily and accurately, reducing the incidence of falls and the accompanying burdens. Dobbels et al. (10) already investigated whether the risk of falling could be predicted using hearing status, sound localization performance, duration of disease, or sport practice but noted that none of these outcomes can be used to distinguish fallers from non-fallers. On the other hand, the Dizziness Handicap Inventory and Oscillopsia Severity Questionnaire did seem to be good predictors of the risk of falling. Schniepp et al. (11) also investigated the potential predictive factors for an increased risk of falls in BVP and noted that an increased temporal gait variability during slow walking was associated with an increased fall risk. Additionally, those patients presenting with concomitant peripheral neuropathy or cerebellar ataxia were also more prone to falls (11, 12).

In addition to assessing the diagnostic criteria, there is a need for outcome measures to assess the balance deficits that these patients encounter (6, 13). The consensus document of the Bárány Society (3) does recommend the Romberg test with eyes open and closed to address balance impairments in BVP. However, concerning the heterogenous representation of symptoms in BVP, such a single test seems inadequate to obtain an accurate representation of the patients' abilities. A recent review on the balance performance of BVP patients (13) indicated the importance of using more advanced and challenging balance tasks in approximating the patients' subjective complaints to enhance the identification of balance deficits and potential future falls.

This study will therefore perform an exploratory investigation of the interrelations between the variables generally included in the work-up of a vestibular patient (i.e., demographic characteristics, vestibular function tests), patient-reported outcome measures related to symptoms and quality of life, clinical balance measures containing more advanced and challenging balance tasks, gait variables, and fall status in BVP patients. By doing so, we can provide useful insights in which outcome measures could potentially be used as predictive factors for falls in BVP, which may thus pave the way to reduce the incidence of falls within this population.

## Methods

### Study Design

This prospective cross-sectional study has been approved by the local Ethics Committee of the University of Antwerp/Antwerp University Hospital (B300201629697). Data collection took place between October 2017 and October 2018 at the Antwerp University Hospital (UZA).

The subjects gave written consent at the time of study inclusion and were aware that data could be used retrospectively for further research. In addition, written informed consent was obtained with regards to publishing indirectly identifiable data.

### Study Participants

A convenient sample of 27 BVP patients was included in this study; these patients were a subset of the patients included in the study of Dobbels et al. (10), recruited from the Otorhinolaryngology, Head, and Neck Surgery Department at the UZA. Only patients who met the diagnostic criteria of bilateral vestibulopathy as proposed by the Barany Society (3) were included in the study: (1) a horizontal angular vestibulo-ocular reflex (VOR) gain <0.6, as measured by the video Head Impulse Test (vHIT) and/or (2) a reduced caloric response (i.e., sum of bithermal, 30 and 44°C, maximum peak slow-phase velocity on each side <6°/s) and/or (3) a reduced horizontal angular VOR gain <0.1 upon sinusoidal stimulation on a rotatory chair.

#### Anthropometric Measurements

For each subject, information concerning age (years), body mass (kilograms), body height (centimeters), and leg length (millimeters) was obtained. Body mass index was calculated based on body mass (kilograms) and body height (meter).

#### Disease-Specific Characteristics

For each subject, information concerning the time since symptom onset (in years) and etiology was collected.

### Vestibular Function Testing

All BVP patients received neuro-otological testing on site when enrolled in the study. The function of the lateral semicircular canal was evaluated at multiple frequencies. All vestibular function tests were performed by experienced examiners (10). Additionally, VOR function was indirectly measured through gaze (in)stability using the Dynamic Visual Acuity test while the subjects were walking on a treadmill (14).

#### Video Head Impulse Testing

The VOR gain, high-frequency function, was determined as the ratio of the area under the eye velocity curve to the head velocity curve from impulse onset to head velocity being zero again (15). Angular head velocity was determined by three mini-gyroscopes; eye velocity was determined by means of an infrared camera recording the right eye. Ten valid head impulses were collected for each semi-circular canal; the target speed for the lateral impulses was >200°/s. Both the mini-gyroscopes and the infrared camera were incorporated in commercially available vHIT goggles (Otometrics, Taastrup, Denmark).

In addition to the lateral semicircular canals, the left anterior and right posterior semicircular canals were assessed during vHIT. This was done by turning the head 40° to the right and applying impulses with a pitch rotation in the plane of the canals. The same procedure was repeated for the right anterior and left posterior semicircular canals, but now with the head rotated 40° to the left. The target speed for the vertical impulses was >150°/s.

#### Rotatory Chair Testing

For determining the low- to mid-frequency function, the subjects were seated in a chair (Servo-Med, Sweden) that was rotated sinusoidally around the Earth's vertical axis by means of a servo-controlled DC motor with a peak angular velocity of 60°/s and a frequency of 0.05 Hz. The total test duration was 2 min, during which the head movement was recorded using an angular rate sensor (Watson type ARS-C152-1A) attached to the subject's head. Eye movements were recorded using ENG by placing disposable Ag/AgCl electrodes (Blue Sensor type N-00-S) medially and laterally to each eye; the electrodes were placed above and below the right eye for vertical eye movement registration. A common ground electrode was placed on the forehead. Impedances <25 kΩ were accepted; differences between electrodes did not exceed 10 kΩ. An eight-channel ENG system (Toennies Nystagliner, Germany) with DC-coupled amplifiers was used, and the test was performed in complete darkness (16). Horizontal angular VOR gain was calculated using the available information.

#### Caloric Testing

The low-frequency function was assessed using bithermal caloric irrigation (16) using air instead of water due to changes in local patient safety guidelines (17). The outer ear canals on both sides were consecutively insufflated with warm (47°C) and cold (26°C) air for 30 s while the subject was positioned supine, with the head inclined at an angle of 30° with the eyes closed. The order of irrigation was warm right, warm left, cold right, and cold left. Using electronystagmography (ENG), nystagmus was recorded as described in subsection “Rotatory Chair Testing.”

#### Saccular Function

Saccular function was assessed by performing c-vestibular evoked myogenic potentials (VEMP) testing as described in Vanspauwen et al. (18). VEMP responses were evoked using 500-Hz tone bursts (rise/fall time = 2 ms; plateau time = 2 ms; repetition frequency = 5.26 Hz) applied with insert earphones (E-A-RTONE Gold, E-A-R, IN, USA). Two consecutive VEMP threshold determinations were performed on each side through lowering the maximal sound intensity [95 dB, normalized hearing level (nHL)] with steps of 5-dB nHL. The subjects were instructed to perform a sternocleidomastoid muscle contraction unilaterally by turning their head opposite to the stimulated side and pushing their check against their hand while in a seated position. The contraction level had to be kept constant at a predefined target level which the subjects could monitor *via* a dial on a computer screen. This target level was defined individually and applied during each VEMP measurement. When no positive peak was seen after 13 ms and no negative peak after 23 ms, the patient was considered to have an absent c-VEMP response (10).

#### Dynamic Visual Acuity Testing

Using the Dynamic Visual Acuity (DVA) test, while walking on a treadmill, all the vestibular sensors are stimulated. Especially the vertical semicircular canals, through a nodding movement of the head, and the otoliths, as a result of the head bouncing up and down, are stimulated in a natural way (19, 20).

Before the DVA test was started, both the test material and the test conditions were controlled as described in Verbecque et al. (14). Revised 2000 Series EDTRS charts with Sloan letters (CDHKNORSVZ) were used. The charts consist of rows of five randomly chosen letters. LogMAR scale and notation were used. The luminance of the illuminated charts was, for the white color, 182.2 cd/m^2^, and for the black color, 3.39 cd/m^2^, with a Weber contrast of 98%. Illumination 0.5 m in front of the chart is 540 lx. The distance between the chart and the subject was 4 m for all measurements, with the chart positioned at eye level. Visual acuity was tested binocularly; subjects who wore glasses or contact lenses could wear them during testing, except for multifocal glasses.

Each subject read the optotypes aloud to determine the visual acuity and started reading at the 0.4 logMAR line. Whenever the subject was unable to read all optotypes correctly, they were instructed to read the line above (0.5 logMAR), which was repeated until all optotypes on the same line were read correctly. Otherwise, the subject had to read lines with decreasing optotype size until >2 optotypes were missed on a single line. Charts with different letter order were used and changed after each condition to avoid recall. Firstly, static visual acuity (SVA) was determined while standing still on the treadmill and keeping their head still. Afterwards, DVA was assessed while walking on the treadmill at 2, 4, and 6 km/h. Test sensitivity for BVP was found to be 97% when combining 2, 4, and 6 km/h (21). The speed conditions were non-randomized, and the subjects were allowed a familiarization period of 1 min for each walking speed. The test procedure was ended prematurely if the subject felt that they could not walk at a higher speed or if the therapist deemed the condition to be unsafe. The subjects were put in a safety harness to avoid falls while standing and walking on the treadmill.

Visual acuity (logMAR) was calculated for both SVA and DVA based on the sum of the number of correctly read letters (TC) and the value of each letter (LV) using the following formula (22):

$$\begin{array}{l} \text{SVA or DVA }\left(\text{logMAR}\right)=1.10-\left(\text{T}{\text{C}}^{*}LV\right) \end{array}$$

Each line consists of five optotypes and represents 0.1 log units; thus, each optotype equals 0.02 log units (LV). Consequently, for a subject to have a normal vision (score of 0), they must be able to correctly read the optotypes of 11 lines, equalling 1.10 log units. A negative value thus indicates a better performance than would be expected.

Subsequently, visual acuity loss (VAL) was determined by subtracting the DVA logMAR value from the SVA value (22):

$$\begin{array}{l} \text{VAL }\left(\text{logMAR}\right)=\text{SVA }\left(\text{logMAR}\right)-\text{DVA }\left(\text{logMAR}\right) \end{array}$$

As a result, whenever this resulted in a negative VAL value, visual acuity was worse during the dynamic condition compared to the static condition. A decrease of ≥0.2 logMAR (i.e., VAL ≤ −0.2) is deemed abnormal at speeds of 2 and 4 km/h, while for 6 km/h, this is ≥0.3 logMAR (1, 21, 23).

### Patient-Reported Outcome Measures

#### Dizziness Handicap Inventory

The Dizziness Handicap Inventory (DHI) is a validated, self-reported questionnaire consisting of 25 items used to determine the self-perceived handicap on emotion, physical, and functional aspects as a consequence of dizziness and instability (24, 25). Each item is scored with “yes” (four points), “sometimes” (two points), or “no” (zero points); total scores range from 0 to 100. A greater self-perceived handicap is indicated by a higher score. Scores between 0 and 30 indicate a mild functional impairment, 31–60 a moderate functional impairment, and 61–100 a severe functional impairment (26).

#### Activities-Specific Balance Confidence Scale

The Activities-Specific Balance Confidence (ABC) scale is used to determine the extent to which persons trust that they can perform various activities of daily life without falling. A total of 16 activities are described, which are scored from 0 to 100%, where 0% means no confidence at all and 100% means maximal confidence in not losing balance during the activity (27). A total score of 1,600 is calculated and subsequently recalculated to a score of 100. A score above 80% indicates a high level of functioning, a score between 80 and 50% indicates a moderate level of functioning, and a score below 50% indicates a low level of functioning (28).

### Clinical Balance Testing

All clinical balance measures were performed by an experienced physical therapist (NH) who was unblinded of the disease status. Where possible, cutoff scores in a population of BVP patients are used. If no such cutoff scores were available, cutoffs for a comparable population (e.g., a vestibulopathic population or, in case this was also not available, an elderly population) were used.

#### Static Balance Testing

Static balance performance was measured using the Static Balance Sum With Eyes Closed (SBS-EC) (29). The SBS-EC consisted of four conditions where the subjects had to stand still with their eyes closed for 30 s. For each condition, the participants had three trials, with the best trial being used in calculating the total score and with the maximal score being 120 s. If a subject was unable to keep standing for 30 s after the third trial, the test was terminated, and the remaining conditions were not performed. The four conditions, all performed with the eyes closed, included (1) standing on firm ground, feet together and clasping the hands while abducting the arms, (2) standing on a 12-cm-thick foam bed measuring 45 by 45 cm and of medium density (60 kg/cm^3^; NeuroCom International Inc., Clackamas, USA), with the feet 5 cm apart, (3) tandem Romberg, standing heel to toe on firm ground, no angle allowed while the arms are free to move, (4) standing on one leg on firm ground, with the arms free to move. The subjects were allowed to choose the leg with which they wanted to stand on and were allowed to alternate between legs between trials in conditions 3 and 4 (29).

In addition to the SBS-EC performance, the AVeCI index was calculated (30). AVeCI stands for the Antwerp Vestibular Compensation Index and uses the SBS-EC performance in combination with the age of the subject:

$$\begin{array}{l} \text{AVeCI}=\;-50+\text{age}\times 0.486+\text{SBS}-\text{EC}\times 0.421 \end{array}$$

This index can be used as a measure for functional balance performance and was validated in a unilateral vestibulopathy population. The more positive the resulting number, the better the performance; the more negative the resulting number, the worse the performance. Up till now, no cutoff scores are available for the SBS-EC or AVeCI.

#### Five-Times-Sit-To-Stand Test

During the Five-Times-Sit-to-Stand Test (FTSST) (31), the subjects start seated with their back against the chair and their arms crossed on their chest. The subjects were instructed to stand up fully and sit down five times as quickly as possible. The examiner started timing when the start sign was given and stopped when the subject's bottom touched the chair on the fifth repetition. The subjects were allowed to place the feet as they wished. A cutoff of 13 s was determined to identify a balance dysfunction in balance or vestibular disorders (31).

#### Timed Up and Go Test

The Timed Up and Go test (TUG) (32) requires the subjects to stand up from a chair, walk 3 m, turn, walk back, and sit down again as quickly and as safely as possible. Subjects who use an assistive device when walking in the community could use this assistive device. The time needed to complete the test was recorded; each subject performed three trials, of which the trial with the lowest time was used in further analyses. In addition to the TUG alone, the subjects performed the TUG with a cognitive task (TUG-C) and the TUG with an upper-extremity motor task (TUG-M) (32). During the TUG-C, the subjects were asked to count backwards in steps of three from a randomly selected number between 80 and 100 while performing the TUG. The TUG-M included carrying a full cup of water while performing the TUG. Subjects needing an assistive device (i.e., a walker, *n* = 2) for ambulation were excluded from the TUG-M condition. For the TUG, a score of ≥13.5 s is indicative for fall risk in BVP patients post-rehabilitation (33). For TUG-C and TUG-M, no specific cutoff scores for fall risk are available for BVP patients; therefore, the cutoff scores for elderly people were used: TUG-C ≥ 15 s and TUG-M ≥ 14.5 s (32).

#### Tinetti Test

The Tinetti test (34) measures balance and gait function and consists of 16 items, subdivided in nine balance-related items and seven gait-related items. The highest achievable score is 28 points, of which 16 can be obtained in the balance component and 12 in the gait component. A higher score indicates a better balance performance, and a lower score indicates a poorer balance performance. No specific cutoff scores are available for the BVP population; therefore, the cutoff scores for an elderly population are used: ≥24/28—low risk of falls, 19–23/28—moderate risk of falls, and ≤ 18/28—high risk of falls (34).

#### Functional Gait Assessment

The Functional Gait Assessment (FGA) (35) is a modified version of the Dynamic Gait Index (DGI), consisting of 10 items and comprising of seven items of the original DGI and three of the new items: “gait with narrow base of support,” “ambulating backwards,” and “gait with eyes closed.” Each item is scored between 0 and 4, with a score of 0 being defined as “severe impairment” and a score of 4 as “normal,” with the maximum score being 30. A lower score indicates a greater functional impairment, with a score of ≤ 22/30 being indicative for an increased fall risk in community-dwelling elderly (35).

### Gait Variables

The gait variables below were measured using an instrumented three-dimensional gait analysis system equipped with eight infrared cameras [Vicon T10, 100 Hz, Vicon Motion Systems Ltd., Oxford, UK, 100 fps, resolution 1 Megapixel (1,120 ×896), three AMTI type OR 6–7 force plates (1,000 fps, 46 × 50 × 8 cm), and one AccuGait® (1,000 fps) force plate]. A more detailed description of the methodology of the biomechanical balance measures described below can be found as Supplementary Material.

#### Spatiotemporal Parameters of Gait

Means and standard deviations (variability) of walking speed (ms^−1^), cadence (steps/min), step time (seconds), length (meter), and width (meter), and the duration of double- and single-support phase (%) were calculated over the total amount of steps recorded during walking at self-selected slow, preferred, and fast walking speeds. The participants were asked to first walk at their self-selected preferred walking speed through the following instruction: “Walk from here to the next mark at your usual walking speed.” Next, the participants were instructed to walk at a slower-than-usual pace: “Walk from here to the next mark slower than you normally would.” Finally, the participants were instructed to walk at a faster-than-usual pace: “Walk from here to the next mark faster than you normally would.” The total amount of steps recorded during slow walking ranged from 6 to 16 steps, during walking at the preferred walking speed from 6 to 16 steps, and during walking at a faster-than-usual pace from 6 to 10 steps.

Although the total number of steps used to calculate the standard deviations seems low, step-to-step variability can be reliably assessed using <15 steps (36, 37). Gait parameters were considered as absolute.

#### Spatial Margins of Stability

The medio-lateral margins of stability (MoS) were defined as the minimum distance between the ankle marker and the extrapolated center of mass (XCoM) along the medio-lateral axis during the single-support phases. The medio-lateral axis was defined as the axis in the transverse plan, perpendicular to the walking direction derived from the center of mass (CoM) coordinates.

The anterior–posterior MoS was defined as the distance between the ankle marker of the leading foot and the XCoM along the anterior–posterior axis at foot touchdown. The anterior–posterior axis is defined as the axis in the transversal plane, parallel to the walking direction derived from the CoM coordinates. The MoS is expressed as an absolute value (mm).

### Fall History

The patients received a questionnaire about falls in the last 12 months (38). This questionnaire was discussed in an interview with the examiner so that all ambiguities were clarified. Based on the fact whether the subject experienced a fall in the last 12 months, they were divided in either the “faller” group or the “non-faller” group. If the subject answered “yes” on the following question, they were considered as a “faller”: “Have you fallen in the past year due to slipping, tripping, and losing balance, thereby ending on the floor or another lower level?” Furthermore, these subjects were asked about (1) how many times they had fallen, (2) where they had fallen, (3) what caused them to fall, and (4) whether they sustained any fall-related injuries.

### Statistical Analysis

Statistical analysis was performed using JMP Pro® statistical software (version 14 for Windows, SAS Institute). To describe the study population, means and standard deviations of subject characteristics, vestibular function testing, clinical patient-reported outcome measures, clinical balance testing, and biomechanical balance measures were calculated for the total study population as for the “fallers” and “non-fallers” subgroups. Due to the small sample size, non-parametric testing was utilized.

To compare categorical data between the “fallers” and “non-fallers” subgroups, Pearson's chi-square test was used; for continuous data, Mann–Whitney *U* test was used. The level of significance was set at 0.05. If Pearson's chi-square test indicated a significant difference between multiple groups (*n* > 2), a *post-hoc* test with Bonferroni corrections was performed.

## Results

### Study Participants

In total, 27 BVP patients were included in this study. The mean age was 58 ± 10 years, with ages ranging between 33 and 74 years. Of the 27 included BVP patients, 10 were female (37%) and 17 were male (63%). In most of the patients (37%), no underlying pathology could be identified; other etiologies included meningitis (17%), Menière's disease (10%), or genetic (10%). Forty-one percent reported a fall in the preceding 12 months. For further analysis, the patients were divided into fallers (*n* = 11) and non-fallers (*n* = 16). No significant differences were found between fallers and non-fallers. All patient characteristics can be found in Tables 1, 2.

**Table 1:** Demographic characteristics of all patients (*n* = 27), fallers and non-fallers.

		**All patients (*n* = 27)**	**Fallers (*n* = 11)**	**Non-fallers (*n* = 16)**	***p*-value**
Sex (*n*, %)	Female	10 (37%)	6 (55%)	4 (25%)	*0.118*
	Male	17 (63%)	5 (45%)	12 (75%)	
Age (mean ± SD; years)		57.9 ± 10.3	57.1 ± 13.1	58.3 ± 8.3	0.827
Age—minimum; maximum (years)		33.4; 74.1	33.4; 74.1	40.1; 72.2	
Body length (mean ± SD; cm)		171.6 ± 10.2	168.8 ± 10.5	171.8 ± 10.1	0.517
Body weight (mean ± SD; kg)		76.32 ± 16.70	75.45 ± 17.32	76.91 ± 16.80	0.716
BMI (mean ± SD; kg/m^2^)		26.01 ± 3.93	26.31 ± 4.20	25.81 ± 3.86	0.790
Leg length (mean ± SD; mm)		817 ± 56	808 ± 51	823 ± 60	0.394
Disease duration (mean ± SD; years)		12.6 ± 11.8	12.3 ± 15.3	12.9 ± 9.30	0.318
Disease duration—minimum; maximum (years)		1; 51	1; 51	3; 38	

*BMI, body mass index; cm, centimeter; kg, kilogram; m, meter; mm, millimeters*.

*The p-values are calculated using Mann–Whitney U test. The italicized value indicates Pearson chi square test result*.

**Table 2:** Patient characteristics with vestibulo-ocular reflex function testing as documented by the diagnostic criteria for bilateral vestibulopathy.

**Subject**	**Age range**	**Time since**	**Faller**	**Etiology**		**Video head impulse**		**Caloric testing (deg/s)**		**Rotatory**
	**(years)**	**onset (years)**				**testing (gain)**				**chair testing (gain)**
				**Left**	**Right**	**Left lateral SCC**	**Right lateral SCC**	**Left**	**Right**	
BVP01	55–60	17	Yes	Menière's disease	Idiopathic	0.14	0.16	0	0	0.01
BVP02	55–60	11	No	Idiopathic		0.78	0.31	8	5	0.02
BVP03	70–75	5	Yes	Idiopathic		0.74	1.01	4	3	0.1
BVP04	65–70	21	No	Meningitis^a^		0.45	0.56	4	5	0.1
BVP05	60–65	7	Yes	Lyme disease		0.41	0.50	13	11	0.08
BVP06	55–60	20	No	Unknown		0.29	0.31	3	2	0.01
BVP07	40–45	17	No	Cerebral malaria^a^		0.88	0.82	0	0	0.04
BVP08	70–75	4	No	Idiopathic		0.27	0.40	0	3	0.05
BVP09	55–60	4	No	Meningitis^a^		0.85	0.98	0	1	0.15
BVP10	40–45	4	Yes	Head trauma		0.88	1.06	11	19	0.05
BVP11	50–55	10	No	Wernicke syndrome		0.13	0.24	5	2	0.21
BVP12	45–50	18	No	Menière's disease		0.13	0.32	0	10	0.07
BVP13	70–75	3	No	Ototoxicity^a^		0.32	0.24	8	0	0.03
BVP14	70–75	6	Yes	Idiopathic		n.a.	n.a.	0	0	0.02
BVP15	60–65	10	No	Head trauma		0.12	0.04	0	0	0.04
BVP16	55–60	3	No	Idiopathic		0.14	0.33	0	0	0.03
BVP17	60–65	6	Yes	Idiopathic	Menière's disease	0.70	0.39	4	0	0.30
BVP18	60–65	38	No	Encephalitis		0.25	0.25	0	0	0.02
BVP19	55–60	6	No	Idiopathic		0.26	0.15	2	0	0.02
BVP20	55–60	7	No	Idiopathic	Resection vestibular schwannoma	0.84	0.38	0	0	0.19
BVP21	50–55	14	No	Idiopathic		0.49	0.92	0	7	0.07
BVP22	30–35	30	Yes	Meningitis		0.56	0.07	0	0	0.06
BVP23	70–75	51	Yes	Ototoxicity		n.a.	n.a.	0	4	0.1
BVP24	45–50	6	Yes	Genetic^a^		n.a.	n.a.	0	0	0.40
BVP25	55–60	1	Yes	Meningitis		0.69	0.42	0	0	0.20
BVP26	45–50	2	Yes	DFNA9		0.89	0.78	0	0	0.05
BVP27	60–65	20	No	DFNA9		0.62	0.61	0	0	0.05

*SCC, semi-circular canal; deg/s, degrees per second*.

*A bilaterally reduced or absent angular vestibulo-ocular reflex (VOR) function has to be documented by video head impulse testing with a bilaterally pathological horizontal VOR gain <0.6 and/or a reduced caloric response (sum of bithermal maximal-peak slow-phase velocity on each side <6 deg/s) and/or a reduced horizontal angular VOR gain <0.1 upon sinusoidal stimulation on a rotatory chair*.

a *Probable etiology*.

### Fall History

In the current sample of 27 BVP patients, 16 patients (59%) reported no falls, one patient reported one fall (4%), two patients reported two falls (7%), and eight patients (30%) reported three or more falls. In most BVP patients that fell, no severe fall-related injuries were reported, except for one patient who suffered a broken hip and broken ribs because of the fall. A more detailed description of the falls and fall-related information can be found in Table 3.

**Table 3:** More detailed description of falls and fall-related information.

	***n***
**Frequency of falls in the preceding 12 months**	
One	1
Two	2
Three or more	8
**Cause of fall**	
Tripping	1
Slipping	1
Dizziness	3
Loss of balance	10
**Place of fall**	
Bed	3
Chair	3
Bath/shower	3
Toilet	3
Stairs	4
Flat surface	5
Garden	5
Street	6
Gutter	3
Public building	4
Getting out of the car	2
Someone else's house	3
**Fall-related injuries**	
Bruises	6
Scrapes	2
Hip fracture	1
Rib fracture	1
Backache	1

### Vestibular Function Test Results

Results of the vHIT, caloric, rotatory chair testing, saccular function, and dynamic visual acuity testing can be found in Table 4. No significant differences were found between fallers and non-fallers for the high-frequency function of the lateral semi-circular canal (SCC) (vHIT) and low- to mid-frequency function (caloric testing, rotatory chair testing).

**Table 4:** Results of the vestibular function tests of both fallers and non-fallers.

	**All patients (*n* = 27)**	**Fallers (*n* = 11)**	**Non-fallers (*n* = 16)**	***p*-value**
**vHIT (high-frequency function)^a^**				
Gain right lateral SCC (mean ± SD)	0.47 ± 0.31	0.55 ± 0.37	0.43 ± 0.28	0.350
Gain left lateral SCC (mean ± SD)	0.49 ± 0.28	0.63 ± 0.25	0.43 ± 0.28	0.106
Sum gain lateral canals (mean ± SD)	0.96 ± 0.54	1.18 ± 0.57	0.86 ± 0.51	0.214
Gain right posterior SCC (mean ± SD)	0.40 ± 0.24	0.51 ± 0.23	0.34 ± 0.23	0.070
Gain left posterior SCC (mean ± SD)	0.49 ± 0.25	0.67 ± 0.18	0.40 ± 0.22	0.009^*^
Sum gain posterior canals (mean ± SD)	0.89 ± 0.45	1.18 ± 0.36	0.73 ± 0.42	0.009^*^
Gain right anterior SCC (mean ± SD)^b^	0.58 ± 0.26	0.64 ± 0.25	0.56 ± 0.27	0.581
Gain left anterior SCC (mean ± SD)^b^	0.58 ± 0.32	0.80 ± 0.19	0.48 ± 0.33	0.026^*^
Sum gain anterior canals (mean ± SD)^b^	1.16 ± 0.48	1.43 ± 0.40	1.04 ± 0.48	0.047^*^
**Caloric testing (low-frequency function)**				
Slow-phase velocity (SPV) bithermal right (mean ± SD; °/s)	2.67 ± 4.52	3.36 ± 6.19	2.19 ± 3.06	0.865
SPV bithermal left (mean ± SD; °/s)	2.30 ± 3.73	2.91 ± 4.79	1.88 ± 2.90	0.827
Sum SPV bilateral bithermal (mean ± SD; °/s)	4.96 ± 7.45	6.27 ± 10.60	4.06 ± 4.37	0.680
**Rotatory chair testing (mid- to low-frequency function)**				
VOR gain (mean ± SD)	0.09 ± 0.09	0.13 ± 0.13	0.07 ± 0.06	0.195
**Saccular function (c-VEMP)**				
Bilaterally absent (*n*)	15	6	9	*0.310*
Unilaterally absent (*n*)	5	1	4	
Bilaterally present (*n*)	1	1	1	
**Dynamic visual acuity testing**				
Static visual acuity (logMAR)	−0.03 ± 0.15	0.02 ± 0.16	−0.07 ± 0.13	0.080
Dynamic visual acuity 2 km/h (logMAR)	0.04 ± 0.15	0.05 ± 0.14	0.03 ± 0.16	0.577
Visual acuity loss 2 km/h (logMAR)	−0.08 ± 0.07	−0.03 ± 0.06	−0.10 ± 0.07	0.034^*^
Dropouts DVA 2 km/h (*n*, %)	0	0	0	*n.a*.
Dynamic visual acuity 4 km/h (logMAR)	0.07 ± 0.15	0.04 ± 0.11	0.08 ± 0.18	0.931
Visual acuity loss 4 km/h (logMAR)	−0.13 ± 0.11	−0.07 ± 0.06	−0.18 ± 0.11	0.013*
Dropouts DVA 4 km/h (*n*, %)	3 (11%)	1 (9%)	2 (13%)	*0.782*
Dynamic visual acuity 6 km/h (logMAR)	0.09 ± 0.14	0.11 ± 0.13	0.07 ± 0.16	0.423
Visual acuity loss 6 km/h (logMAR)	−0.17 ± 0.11	−0.15 ± 0.11	−0.20 ± 0.11	0.277
Dropouts DVA 6 km/h (*n*, %)	10 (37%)	3 (27%)	7 (44%)	*0.384*

*SCC, semicircular canal; °/s, degrees per second; km/h: kilometers per hour; DVA, dynamic visual acuity; n.a., not available*.

The p-values were calculated using Mann–Whitney U test. The italicized values indicate Pearson chi square test result.

a *vHIT data of three fallers are missing due to malfunctioning of the vHIT goggles*.

b vHIT data of one additional faller and one additional non-faller are missing due to goggle fit issues.

* *p < 0.05*.

Although not significant (*p* = 0.214), non-fallers (0.86 ± 0.13) showed lower vHIT gains for both lateral canals combined as compared to fallers (1.27 ± 0.61). The sum of gains of the left and right posterior and the left and right anterior SCCs, however, did differ between fallers and non-fallers. For the sum of both posterior canals, fallers presented with a higher gain as compared to non-fallers: 1.18 ± 0.36 vs. 0.73 ± 0.42 (*p* = 0.009). The same was noted for the sum of gains of both anterior SCCs, with a gain of 1.43 ± 0.40 for fallers and 1.04 ± 0.48 for non-fallers (*p* = 0.047).

The bilateral bithermal caloric responses of fallers tended to be higher compared to the responses of non-fallers: 6.27 ± 10.60 vs. 4.06 ± 4.37°/s (*p* = 0.680). The same trend was noted for the VOR gain during rotatory chair testing: 0.07 ± 0.06 for non-fallers vs. 0.13 ± 0.13 for fallers (*p* = 0.195).

Saccular function was assessed with c-VEMP testing; in 15 patients (71%) no c-VEMP response could be evoked bilaterally, in five patients (24%) a unilateral response was present, and in one patient (5%) responses were found bilaterally. In six patients, the c-VEMP test results were not available. The chi-square test results did not show significant differences between saccular function and falls (*p* = 0.310). However, the proportion of patients with a bilaterally absent c-VEMP response was higher for fallers.

**Figure 1:**
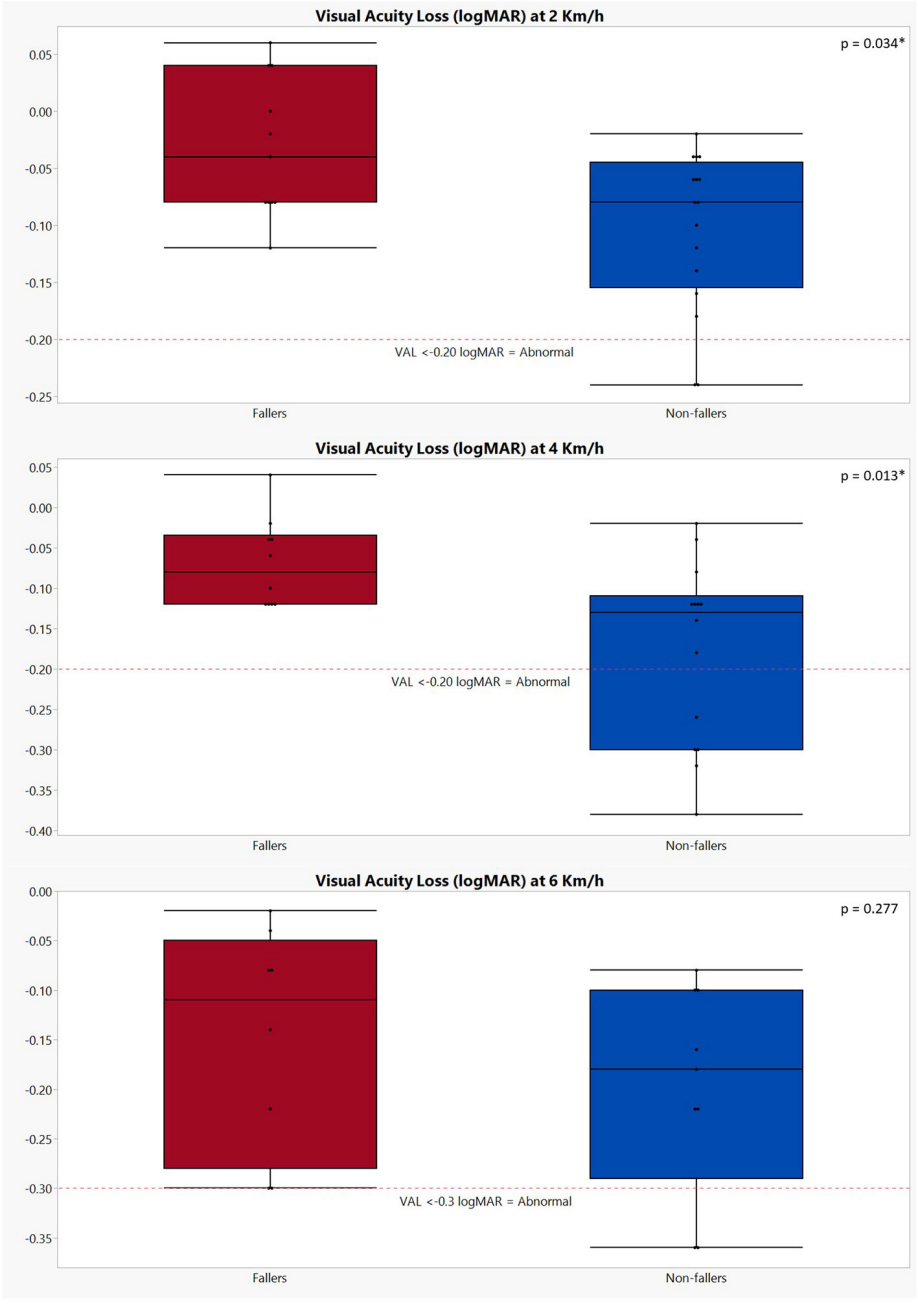
Graphical representation of the visual acuity loss during dynamic visual acuity testing on the treadmill in both fallers and non-fallers. The performances of fallers are indicated in red; the performances of non-fallers are indicated in blue. VAL, visual acuity loss. The *p*-values were calculated using Mann–Whitney *U* test; *p < 0.05. Number of subjects completing the DVA testing at 2 km/h: fallers: *n* = 11; non-fallers: *n* = 16; 4 km/h: fallers: *n* = 10; non-fallers: *n* = 14; 6 km/h: fallers: *n* = 8; non-fallers: *n* = 9. A decrease of ≥0.2 logMAR (i.e., VAL ≤ −0.2) is deemed abnormal at speeds of 2 and 4 km/h, while for 6 km/h, this is ≥0.3 logMAR (1, 21, 23).

Lastly, results of the dynamic visual acuity testing during treadmill walking revealed that, although not significant (*p* = 0.080), non-fallers had better static visual acuity (−0.07 ± 0.13) as compared to fallers (0.02 ± 0.16). However, significant differences were found in visual acuity loss when walking at 2 km/h (*p* = 0.034) and 4 km/h (*p* = 0.013), where non-fallers showed a greater loss of visual acuity (VAL 2 km/h: −0.10 ± 0.07 logMAR; VAL 4 km/h: −0.18 ± 0.11 logMAR) compared to the fallers (VAL 2 km/h: −0.03± 0.06 logMAR; VAL 4 km/h: −0.07 ± 0.06 logMAR). For the visual acuity loss at 6 km/h, the same trend was noted but failed to reach significance (non-fallers: −0.20 ± 0.11 logMAR; fallers: −0.15 ± 0.11 logMAR; *p* = 0.277) (Figure 1). Additionally, although the proportion of dropouts for the 4- and 6-km/h conditions in the group of non-fallers was higher, the chi-square test results did not show any significant differences [*p* = (0.384, 0.782)].

### Patient-Reported Outcome Measures

**Figure 2:**
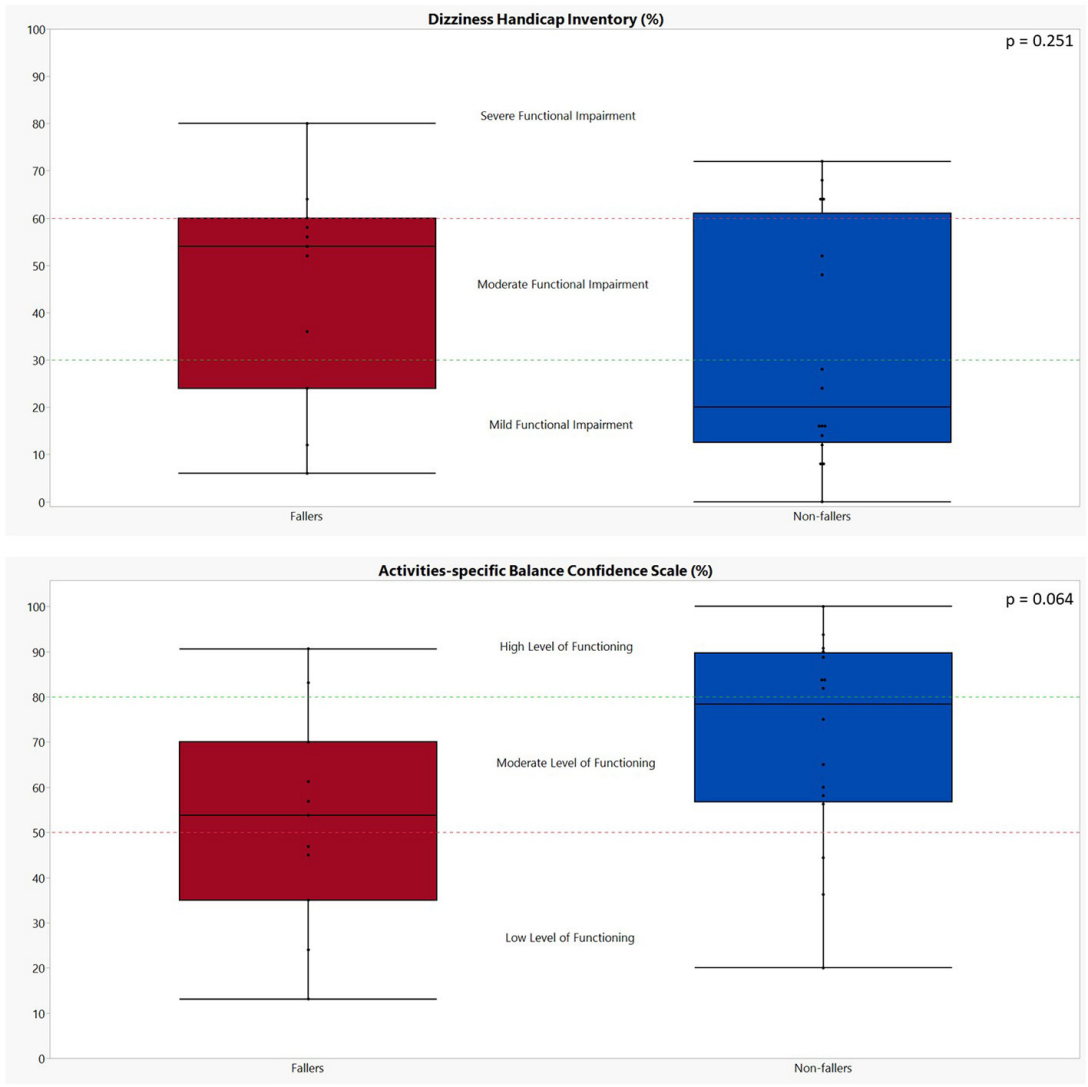
Graphical representation of the performances of both fallers and non-fallers on the patient-reported outcome measures. The performances of fallers (*n* = 11) are indicated in red; the performances of non-fallers (*n* = 16) are indicated in blue. The *p*-values were calculated using Mann–Whitney *U* test. Dizziness Handicap Inventory: scores between 0 and 30 indicate a mild functional impairment, 31 to 60 indicate a moderate functional impairment, and 61 to 100 indicate a severe functional impairment (26). Activities-Specific Balance Confidence scale: a score above 80% indicates a high level of functioning, a score between 80 and 50% indicates a moderate level of functioning, and a score below 50% indicates a low level of functioning (28).

All results of the patient-reported outcome measures (PROMs) can be found in Table 5 and Figure 2. In general, fallers tended to have a worse self-perceived functional impairment due to dizziness (DHI; 46 ± 23 vs. 32 ± 25 for non-fallers); however, the results were not significant (*p* = 0.251). Only the physical subscale of the DHI showed a statistically significant difference between fallers and non-fallers (17 ± 5 vs. 9 ± 7, *p* = 0.006). In addition, although not significant, a greater percentage (72.73%) of fallers experienced moderate to severe functional impairments as compared to non-fallers (37.50%). For the ABC scores, the fallers also showed less confidence in performing activities of daily life when compared to non-fallers, but again these differences were non-significant: 52.69 ± 23.56% for fallers vs. 70.47 ± 23.00% for non-fallers (*p* = 0.064). Based on the ABC scores, a greater percentage of fallers (81.82%) were defined as having a moderate- to low-level functioning as compared to 50% of non-fallers. However, these differences also failed to reach significance (*p* = 0.183).

**Table 5:** Results of the patient-reported outcome measures [Dizziness Handicap Inventory (DHI) and Activities-Specific Balance Confidence (ABC) scale] of all patients and both fallers and non-fallers.

	**All patients (*n* = 27)**	**Fallers (*n* = 11)**	**Non-fallers (*n* = 16)**	***p*-value**
**Dizziness Handicap Inventory (mean** **±** **SD)**				
Emotion subscale (…/36)	10 ± 9	12 ± 9	9 ± 8	0.368
Physical subscale (…/24)	12 ± 7	17 ± 5	9 ± 7	0.006^*^
Functional subscale (…/40)	16 ± 12	17 ± 11	15 ± 12	0.544
Total score (…/100)	38 ± 25	46 ± 23	32 ± 25	0.251
Mild functional impairment (*n*, %)	13 (48.15%)	3 (27.27%)	10 (62.50%)	*0.058*
Moderate functional impairment (*n*, %)	8 (29.63%)	6 (54.55%)	2 (12.50%)	
Severe functional impairment (*n*, %)	6 (22.22%)	2 (18.18%)	4 (25.00%)	
**Activities-Specific Balance Confidence scale (mean** **±** **SD)**				
ABC total (…/100%)	63.23 ± 24.45	52.69 ± 23.56	70.47 ± 23.00	0.064
High-level functioning (*n*, %)	10 (37.04%)	2 (18.18%)	8 (50.00%)	*0.183*
Moderate-level functioning (*n*, %)	9 (33.33%)	4 (36.36%)	5 (31.25%)	
Low-level functioning (*n*, %)	8 (29.63%)	5 (45.45%)	3 (18.75%)	

*Mild functional impairment DHI: 0, 30; moderate functional impairment DHI: 31, 60; severe functional impairment DHI: 31, 100; high-level functioning ABC: 81, 100%; moderate-level functioning ABC: 50, 80%; low-level functioning ABC: 0, 50%*.

*The p-values were calculated using Mann–Whitney U test. The italicized values indicate Pearson chi-square test result*.

* *p < 0.05*.

### Clinical Balance Testing

**Figure 3:**
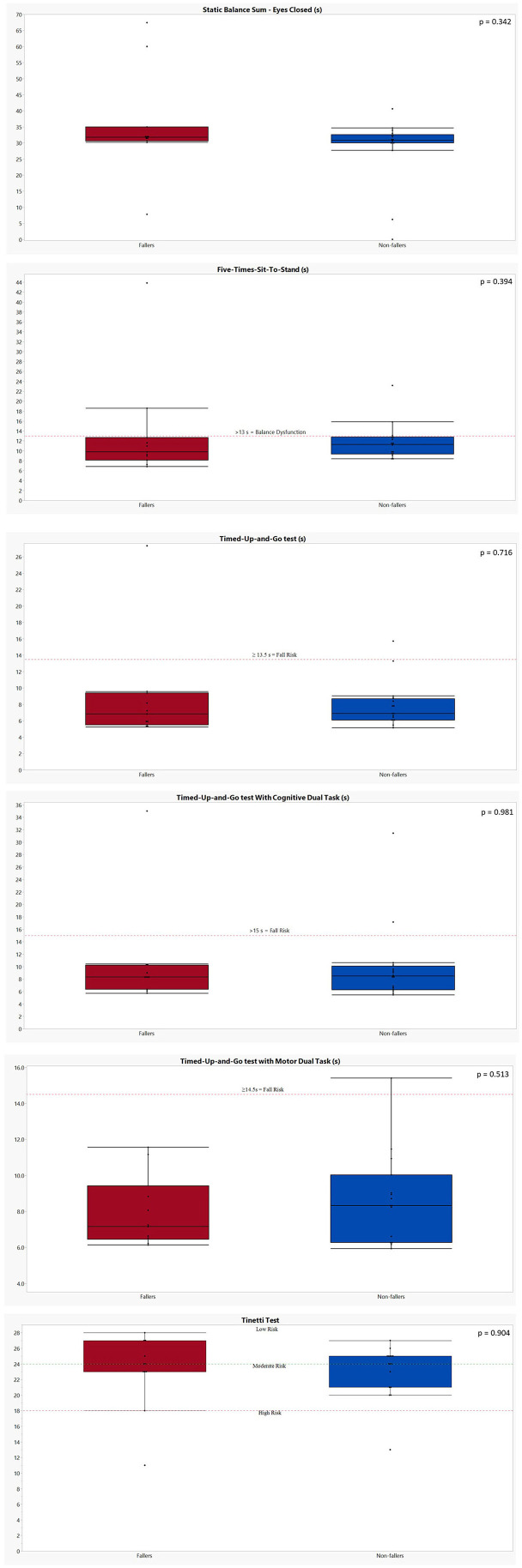
Graphical representation of the performances of both fallers and non-fallers on the clinical balance tests. The performances of fallers (*n* = 11) are indicated in red; the performances of non-fallers (*n* = 16) are indicated in blue. The *p*-values were calculated using Mann–Whitney *U* test. (1) Static balance sum—eyes closed: no cutoff values available. Five-Times-Sit-to-Stand: a cutoff of 13 s is indicative for a balance dysfunction in balance or vestibular disorders (31). (2) Timed Up and Go test: a score of ≥13.5 s is indicative for fall risk in patients with bilateral vestibulopathy post-rehabilitation (33). Timed Up and Go test with cognitive dual task: a score of ≥15 s is indicative for fall risk in an elderly population (32). (3) Timed Up and Go test with motor dual task: one faller and one non-faller were unable to perform the TUG-M due to their need for an assistive device. A score of ≥14.5 s is indicative for fall risk in an elderly population (32). Tinetti test: a score of ≥24/28 indicates a low risk of falls; 19–23/28: moderate risk of falls; ≤18/28: high risk of falls in an elderly population (34). (4) Functional gait assessment: A score of ≤ 22/30 is indicative for an increased fall risk in community-dwelling elderly (35).

The results of all clinical balance tests can be found in Table 6 and Figure 3 (1–4). No significant differences could be found between fallers and non-fallers on any of the balance tests. The group of fallers even showed a better performance on the SBS-EC compared to the non-fallers: 35.41 ± 15.84 vs. 28.41 ± 10.34 s (*p* = 0.342). This resulted in non-fallers having (−9.67 ± 5.17) a lower score on the AVeCI index as compared to the fallers (−7.35 ± 6.59; *p* = 0.312). On the FTSST, although not significant, fallers needed a longer time to complete the five repetitions: 13.41 ± 10.60 vs. 11.73 ± 3.65 s for non-fallers (*p* = 0.394). Concerning the TUG and TUG combined with a cognitive or motor task, fallers performed slightly, but not significantly, worse on the TUG (8.76 ± 6.35 vs. 7.91 ± 2.85 s; *p* = 0.716) and TUG-C (10.55 ± 8.26 vs. 9.95 ± 6.40 s; *p* = 0.981) but performed slightly better on the TUG-M (7.94 ± 1.99 vs. 8.71 ± 2.54; *p* = 0.531). For both the Tinetti subscores and total score, as the FGA, the performances of fallers and non-fallers did not differ (*p* = 0.790, 0.904).

**Table 6:** Results of the clinical balance tests of all patients and both fallers and non-fallers.

	**All patients (*n* = 27)**	**Fallers (*n* = 11)**	**Non-fallers (*n* = 16)**	***p*-value**
**Static balance sum with eyes closed (mean** **±** **SD)**				
Seconds (…/120)	31.26 ± 13.06	35.41 ± 15.85	28.41 ± 10.34	0.342
AVeCI	−8.74 ± 5.79	−7.35 ± 6.59	−9.69 ± 5.17	0.312
**Five-Times-Sit-to-Stand Test (mean** **±** **SD)**				
Seconds	12.42 ± 7.19	13.41 ± 10.60	11.73 ± 3.65	0.394
**Timed Up and Go (TUG) test (mean** **±** **SD)**				
TUG (seconds)	8.26 ± 4.51	8.76 ± 6.35	7.91 ± 2.85	0.716
TUG—cognitive (seconds)	10.19 ± 7.07	10.55 ± 8.26	9.95 ± 6.40	0.981
TUG—motor (seconds)^a^	8.40 ± 2.32	7.94 ± 1.99	8.71 ± 2.54	0.531
**Tinetti test (mean** **±** **SD)**				
Tinetti balance (…/16)	13 ± 2	13 ± 3	13 ± 2	0.904
Tinetti gait (…/12)	10 ± 2	10 ± 2	10 ± 2	0.544
Tinetti total (…/28)	23 ± 4	23 ± 5	23 ± 3	0.904
**Functional gait assessment (mean** **±** **SD)**				
Total score (…/30)	18 ± 6	18 ± 5	19 ± 6	0.790

*SD, standard deviation; TUG, Timed Up and Go test*.

a *One faller and one non-faller did not perform the TUG with upper extremity motor task due to the use of a walker*.

*The p-values were calculated using Mann–Whitney U test*.

### Gait Variables

Table 7 contains the results of both the spatiotemporal gait parameters and the margins of stability at self-selected slow, preferred, and fast walking speeds of all BVP patients.

**Table 7:** Means and standard deviations (variability) of the spatiotemporal gait parameters and spatial margins of stability at all walking speeds in all bilateral vestibulopathy patients.

	**Slow walking**			**Preferred walking**			**Fast walking**		
	**Mean**	**SD**	**95% CI**	**Mean**	**SD**	**95% CI**	**Mean**	**SD**	**95% CI**
**Gait parameters**									
Walking speed (m/s)	0.81	0.17	0.73, 0.88	1.25	0.18	1.18, 1.33	1.58	0.27	1.47, 1.70
Cadence (steps/min)	96	11	91, 101	122	9	118, 126	136	15	129, 142
Step time (s)	0.63	0.08	0.60, 0.67	0.50	0.04	0.48, 0.51	0.45	0.05	0.43, 0.47
Step length (m)	0.52	0.07	0.49, 0.55	0.62	0.09	0.58, 0.66	0.72	0.10	0.67, 0.76
Step width (m)	0.18	0.05	0.16, 0.20	0.18	0.04	0.16, 0.20	0.18	0.05	0.16, 0.20
Single-support phase (%)	36.92	2.35	35.90, 37.93	39.54	2.03	38.66, 40.41	41.95	1.91	41.12, 42.78
Double-support phase (%)	26.03	4.61	24.03, 28.02	20.87	3.89	19.18, 22.55	15.92	3.59	14.37, 17.47
Walking speed SD (m/s)	0.03	0.01	0.02, 0.04	0.05	0.03	0.04, 0.06	0.03	0.02	0.03, 0.04
Cadence SD (steps/min)	5	3	4, 6	5	4	3, 7	4	2	3, 6
Step time SD (s)	0.03	0.02	0.02, 0.04	0.02	0.01	0.01, 0.03	0.01	0.01	0.01, 0.02
Step length SD (m)	0.03	0.01	0.02, 0.03	0.03	0.02	0.02, 0.04	0.03	0.01	0.02, 0.03
Step width SD (m)	0.03	0.02	0.02, 0.04	0.03	0.02	0.02, 0.04	0.03	0.01	0.03, 0.04
Single-support phase SD (%)	2.23	1.03	1.79, 2.68	1.50	0.94	1.10, 1.91	1.47	0.65	1.19, 1.75
Double-support phase SD (%)	2.16	0.80	1.81, 2.50	1.48	0.78	1.45, 1.82	1.43	0.60	1.17, 1.69
**Spatial margins of stability**									
AP MoS (mm)	−44	42	−63, −27	−147	42	−165, −129	−238	68	−267, −209
ML MoS (mm)	45	13	39, 50	50	15	43, 57	48	19	39, 56

*m/s, meter per second; steps/min, steps per minute; s, second; m, meter; %, percentage of gait cycle; SD, standard deviation; mm, millimeter; AP MoS, anterior–posterior margin of stability; ML MoS, medio-lateral margin of stability*.

*The p-values were calculated using Mann–Whitney U test*.

None of the included parameters during walking at preferred walking speed (Table 9) showed a significant difference between fallers and non-fallers (*p* = 0.284, 1.000). Although not significant (*p* = 0.343), fallers tended to walk slightly faster (1.31 ± 0.16 m/s) as compared to the non-fallers (1.21 ± 0.18 m/s). In general, fallers did show less variability as compared to non-fallers, although none of the variability parameters was deemed as significantly different (Table 9). For the margins of stability, fallers showed a decreased AP MoS (−159 ± 52 vs. −138 ± 32 mm for non-fallers; *p* = 0.313); ML MoS did not differ (49 ± 14 mm vs. 51 ± 17 for non-fallers; *p* = 0.879).

In Table 8, a comparison of the included gait parameters and MoS between fallers and non-fallers at slow walking speed can be found. No significant differences were found regarding the spatiotemporal gait parameters (*p* = 0.232, 1.000) or MoS (*p* = 0.313, 0.976).

**Table 8:** Means and standard deviations (variability) of the spatiotemporal gait parameters and spatial margins of stability at slow walking speed in the fallers and non-fallers bilateral vestibulopathy patients.

	**Fallers (*****n*** **=** **10)**			**Non-fallers (*****n*** **=** **13)**			***p*-value**
	**Mean**	**SD**	**95% CI**	**Mean**	**SD**	**95% CI**	
**Gait parameters**							
Walking speed (m/s)	0.84	0.15	0.73, 0.94	0.79	0.19	0.67, 0.90	0.483
Cadence (steps/min)	98	11	90, 106	95	11	88, 101	0.410
Step time (s)	0.62	0.09	0.56, 0.69	0.64	0.07	0.60, 0.69	0.410
Step length (m)	0.53	0.05	0.49, 0.56	0.52	0.09	0.56, 0.57	1.000
Step width (m)	0.16	0.04	0.14, 0.19	0.19	0.05	0.16, 0.22	0.232
Single-support phase (%)	37.50	2.69	35.58, 39.43	36.47	2.05	35.23, 37.71	0.784
Double-support phase (%)	24.95	5.21	21.23, 28.67	26.86	4.12	24.37, 29.34	0.738
Walking speed SD (m/s)	0.03	0.01	0.02, 0.04	0.03	0.02	0.02, 0.04	0.522
Cadence SD (steps/min)	5	2	4, 6	5	3	3, 7	0.605
Step time SD (s)	0.03	0.02	0.02, 0.05	0.04	0.02	0.02, 0.05	0.784
Step length SD (m)	0.03	0.01	0.02, 0.04	0.03	0.02	0.02, 0.04	0.693
Step width SD (m)	0.03	0.02	0.02, 0.05	0.03	0.01	0.02, 0.03	0.343
Single-support phase SD (%)	2.14	0.93	1.47, 2.80	2.31	1.32	1.62, 2.99	1.000
Double-support phase SD (%)	1.95	0.76	1.41, 2.50	2.32	0.82	1.82, 2.81	0.313
**Spatial margins of stability**							
AP MoS (mm)	−54	51	−91, −17	−38	35	−59, −17	0.313
ML MoS (mm)	44	12	36, 53	45	14	36, 54	0.976

*m/s, meter per second; steps/min, steps per minute; s, second; m, meter; %, percentage of gait cycle; SD, standard deviation; mm, millimeter; AP MoS, anterior–posterior margin of stability; ML MoS, medio-lateral margin of stability*.

*The p-values were calculated using Mann–Whitney U test*.

**Table 9:** Means and standard deviations (variability) of the spatiotemporal gait parameters and spatial margins of stability at preferred walking speed in faller and non-faller bilateral vestibulopathy patients.

	**Fallers (*****n*** **=** **10)**			**Non-fallers (*****n*** **=** **13)**			***p*-value**
	**Mean**	**SD**	**95% CI**	**Mean**	**SD**	**95% CI**	
**Gait parameters**							
Walking speed (m/s)	1.31	0.16	1.20, 1.43	1.21	0.18	1.10, 1.32	0.343
Cadence (steps/min)	122	7	117, 128	121	11	115, 127	0.693
Step time (s)	0.49	0.03	0.47, 0.51	0.50	0.04	0.48, 0.53	0.648
Step length (m)	0.64	0.06	0.60, 0.68	0.61	0.11	0.54, 0.68	0.522
Step width (m)	0.18	0.04	0.15, 0.20	0.18	0.04	0.16, 0.21	0.832
Single-support phase (%)	39.79	1.64	38.61, 40.96	39.34	2.33	37.93, 40.75	1.000
Double-support phase (%)	20.14	3.25	17.82, 22.47	21.42	4.36	18.79, 24.06	1.000
Walking speed SD (m/s)	0.04	0.02	0.03, 0.06	0.06	0.03	0.04, 0.08	0.343
Cadence SD (steps/min)	4	2	2, 6	6	4	3, 8	0.446
Step time SD (s)	0.02	0.01	0.01, 0.02	0.02	0.02	0.01, 0.03	0.284
Step length SD (m)	0.02	0.02	0.01, 0.04	0.03	0.03	0.02, 0.05	0.522
Step width SD (m)	0.03	0.01	0.02, 0.04	0.03	0.02	0.02, 0.04	0.693
Single-support phase SD (%)	1.34	0.82	0.76, 1.93	1.62	1.03	1.00, 2.25	0.605
Double-support phase SD (%)	1.39	0.58	0.98, 1.80	1.55	0.92	1.00, 2.11	0.927
**Spatial margins of stability**							
AP MoS (mm)	−159	52	196, −122	−138	32	−157, −118	0.313
ML MoS (mm)	49	14	39, 58	51	17	41, 61	0.879

*m/s, meter per second; steps/min, steps per minute; s, second; m, meter; %, percentage of gait cycle; SD, standard deviation; mm, millimeter; AP MoS, anterior–posterior margin of stability; ML MoS, medio-lateral margin of stability*.

*The p-values were calculated using Mann–Whitney U test*.

Table 10 contains the spatiotemporal parameters and MoS during the fast walking trial of both fallers and non-fallers. No significant differences were found here as well between fallers and non-fallers concerning the spatiotemporal parameters (*p* = 0.343, 0.976) or the MoS (*p* = 0.605, 0.879).

**Table 10:** Means and standard deviations (variability) of the spatiotemporal gait parameters and spatial margins of stability at fast walking speed in the faller and non-faller bilateral vestibulopathy patients.

	**Fallers (*****n*** **=** **10)**			**Non-fallers (*****n*** **=** **13)**			***p*-value**
	**Mean**	**SD**	**95% CI**	**Mean**	**SD**	**95% CI**	
**Gait parameters**							
Walking speed (m/s)	1.59	0.19	1.45, 1.73	1.58	0.32	1.39, 1.78	0.976
Cadence (steps/min)	137	12	129, 145	135	17	124, 145	0.343
Step time (s)	0.44	0.04	0.41, 0.47	0.45	0.05	0.42, 0.48	0.343
Step length (m)	0.71	0.08	0.66, 0.77	0.72	0.12	0.65, 0.79	0.648
Step width (m)	0.17	0.02	0.15, 0.18	0.18	0.06	0.15, 0.22	0.343
Single-support phase (%)	42.19	1.82	40.89, 43.49	41.76	2.03	40.53, 42.99	0.648
Double-support phase (%)	15.67	3.58	13.13, 18.25	16.10	3.72	13.86, 18.35	0.784
Walking speed SD (m/s)	0.03	0.02	0.02, 0.05	0.03	0.02	0.02, 0.05	0.605
Cadence SD (steps/min)	4	1	3, 5	4	2	3, 6	0.879
Step time SD (s)	0.01	0.01	0.01, 0.02	0.02	0.01	0.01, 0.02	0.648
Step length SD (m)	0.03	0.01	0.02, 0.03	0.03	0.02	0.2, 0.04	0.927
Step width SD (m)	0.03	0.01	0.02, 0.03	0.03	0.02	0.02, 0.04	0.976
Single-support phase SD (%)	1.40	0.68	0.92, 1.89	1.52	0.65	1.13, 1.91	0.879
Double-support phase SD (%)	1.52	0.59	1.09, 1.94	1.36	0.61	0.99, 1.73	0.563
**Spatial margins of stability**							
AP MoS (mm)	−242	53	−280, −203	−235	79	−283, −187	0.605
ML MoS (mm)	47	12	38, 56	48	24	34, 63	0.879

*m/s, meter per second; steps/min, steps per minute; s, second; m, meter; %, percentage of gait cycle; SD, standard deviation; mm, millimeter; AP MoS, anterior–posterior margin of stability; ML MoS, medio-lateral margin of stability*.

*The p-values were calculated using Mann–Whitney U test*.

## Discussion

The aim of the present study was to explore potential relationships between demographic characteristics, clinical measures, patient-reported measures and gait variables, and fall status in BVP patients. The results of the current study indicate that none of the included outcome measures seem useful to distinguish between those BVP patients who fell in the preceding 12 months and those who did not. Although the DHI and ABC showed a tendency to be worse in fallers, the differences did not reach significance. This emphasizes the need for a more thorough investigation concerning the predictive ability of outcome measures for BVP patients' fall status.

The included BVP population is comparable and representative to the BVP populations included in previous studies in terms of age and gender (39–43). The prevalence of falls in the present cohort is also comparable with those of previous studies investigating fall status in BVP patients (8, 11, 41, 42, 44). The previously reported fall prevalence ranged between 38% in the previous 6 months (11) and 55% since disease onset (42), while in the present study the prevalence of falls was 41% over the past 12 months.

### Vestibular Function Tests and Fall Risk

None of the included vestibular function tests related to the Bárány diagnostic criteria of BVP (i.e., vHIT of the lateral SCC, calorics, rotatory chair) (3) displayed differences between fallers and non-fallers in this population of BVP patients. This result is in line with previous reports, where Schniepp et al. (11) also did not find any correlation between caloric responses and falls in BVP patients. However, by only including function tests of the lateral SCC, potentially crucial information on anterior and posterior SCC and otolithic function is missed (10, 42). As anterior and posterior SCC function was also included in the current test protocol, this provides additional insights in the vestibular functioning of BVP patients. The current results indicate an increased sparing of the high-frequency function of the anterior and posterior SCCs in the fallers subgroup. These results are comparable to those of Dobbels et al. (10), where a significant increase in gain of the posterior SCC was also found; although the same trend was found for the anterior SCC, significance was lost.

In addition, it must be stipulated that ~29% of BVP patients, where c-VEMP testing was possible, presented with at least a unilateral sparing of saccular function. The consequences due to loss of otolithic functioning, however, are still unclear, although BVP patients often present with an altered saccular and utricular function (42, 45, 46). Agrawal et al. (46), for example, did find a greater association between saccular dysfunction (cVEMP amplitudes) and subjective functional impairment, as determined by the DHI, compared to SCC dysfunction. Additionally, previous reports of BVP patients presenting with solely an impaired otolith function also reflect that a wide variety in vestibular impairment is present within the BVP population itself (45, 47). Therefore, it seems sensible that a single test evaluating vestibular function is not capable of distinguishing fallers from non-fallers. As Dobbels et al. (10) have suggested earlier, differentiating between the different patterns of vestibular impairments present in the BVP population and linking those with fall risk may provide the clinicians or healthcare professionals more insights in the identification of those patients at an increased risk of falling.

To summarize, although BVP patients can present with a limited vestibular impairment based on different vestibular function tests, they can still be prone to falls. Therefore, based on the information presented above, we suggest that vestibular function tests should rather be used to evaluate the vestibular system itself, with the purpose of making diagnosis rather than determining the fall risk.

In contrast to the vestibular function tests described by the diagnostic criteria (3), which only stimulate the lateral semi-circular canals, the dynamic visual acuity testing during walking on the treadmill is a more functional outcome as it comprises an active movement, stimulating all semi-circular canals in addition to the otoliths at the same time (48). As a result, those patients with an increased residual function of the vestibular system may thus be able to use this information more efficiently, resulting in a smaller visual acuity loss during walking. This may be a possible explanation for the results found in the current study. As the group of fallers displayed an increased residual function, based on the different vestibular function tests, this may explain the smaller loss of visual acuity during the three speed conditions (i.e., 2, 4, and 6 km/h). The number of dropouts for each condition was comparable to the rates reported by previous studies. In this study and the majority of other studies (21, 49) reporting DVA testing during treadmill walking, no patients dropped out during walking at 2 km/h. Only the study of van Dooren et al. (50) reported a dropout of 9% at 2 km/h. For DVA testing at 4 km/h, the dropout rates ranged between 5% (21) and 14% (49), while in the present study this was 11%. However, the majority of dropouts in the present study were found in the group of non-fallers. For the 6-km/h condition, the dropout rates ranged between 22% (21) and 48% (50), while in the present study this was 37%. The majority of dropouts here were situated in the non-faller group as well. A possible explanation may be, again, the increased sparing of the sensory function of the vestibular system in the fallers. As the DVA test is a functional outcome where the different sensory systems are in play due to integration of the visual, vestibular, and oculomotor systems, an increased residual function may facilitate the adaptation and compensation mechanisms useful during (treadmill) walking (49, 50).

### PROMs and Fall Risk

Scores on the DHI indicate that about 50% of the BVP patients suffer from a moderate to severe functional impairment. This percentage is lower than previously reported results on the DHI. Guinand et al. (51) found that 85% of the BVP patients had a moderate to severe functional impairment, with 44% of those patients perceiving their handicap as severe. Hermann et al. (42) also reported that 85% of the included BVP patients indicated to have a moderate (60%) to severe (25%) functional impairment. No significant differences were found between fallers and non-fallers on the total DHI score in the present study. Hermann et al. (42), however, did find a significant difference in DHI scores between fallers and non-fallers. Dobbels et al. (10) also found a significant difference between fallers and non-fallers on the DHI and all its subscales. This may indicate that the present sample size with 11 fallers and 16 non-fallers is too small to detect significant differences in total DHI scores. On the other hand, it should be noted that 73% of the fallers were classified as having a moderate to severe functional impairment as compared to 38% of the non-fallers, so even if no difference was found on total DHI scores, in general, fallers tended to have a higher self-perceived handicap.

As for the confidence in performing certain activities of daily life, the mean ABC score (63%) was comparable to the score reported by Schniepp et al. (11) of 65%, indicating a moderate level of functioning. No significant differences were noted here as well between fallers and non-fallers, although fallers reported a lower level of functioning than the non-faller group. A total of 81% of the included BVP patients that fell were classified under the moderate- and low-level functioning category, with 45% being classified as low-level functioning. On the other hand, 50% of non-fallers were classified as moderate- to low-level functioning, with only 19% of non-fallers indicating as having low-level functioning.

Although neither the DHI nor the ABC was able to distinguish between fallers and non-fallers in the present population, PROMs may be valuable tools to determine the subjective experience of the patient. The results above could indicate that BVP fallers who have fallen in the past have adapted their behavior in daily life in order to limit the risk of falling again. However, a more thorough investigation with a large sample is suggested to further determine the potential discriminating ability of these PROMs.

### Clinical Balance Measures and Fall Risk

Information on balance performance of BVP patients on the included clinical balance measures is lacking in currently existing literature, as indicated by a recent review on this matter (13). Especially information on the performance of fallers compared to non-fallers is scarce.

Most studies which reported standing balance performance of BVP patients reported either center of pressure (CoP) measures (e.g., amplitude, area, and velocity), dynamic alignment (i.e., angular position of the CoP relative to the base of support), or strategy (i.e., amount of ankle and hip movements) (13), all of which require specialized and expensive equipment. A simple time measure, on the other hand, does not require specialized equipment and can be done anywhere. Concerning the current results on standing balance (SBS-EC), most of the patients were able to perform the standing-with-feet-together-with-eyes-closed condition; however, they failed to keep on standing in the standing-on-foam-with-eyes-closed condition. Surprisingly, fallers tended to show a better performance than non-fallers, although this difference was not significant. A possible explanation may be the slightly better results on the rotatory chair test, present in the faller group. As BVP patients substitute the vestibular loss with a combination of proprioceptive and visual cues to compensate the missing vestibular information, a better, although still impaired, vestibular function may be beneficial in situations involving multiple sensory perturbations (e.g., standing on foam with eyes closed). When comparing the current scores of BVP patients on the AVeCI index (−8.70) to the scores of subjects with a normal vestibular function (7.25), patients with unilateral vestibular loss who are compensated (4.18), and patients with unilateral vestibular loss who are uncompensated (−6.42) (30), the AVeCI seems useful in determining functional balance performance in BVP.

An impaired ability to move from a seated position to a standing position can significantly limit the functional abilities of a person. Therefore, the FTSST is used to assess the functional strength of the lower limbs, balance, performance of translation movements, and fall risk (31). In the current sample, 12 falls were related to places where a transfer from one position to another is performed (e.g., bed, chair, bath, or toilet). Considering the cutoff score of 13 s (31), the total population would not be considered as having a balance dysfunction. When considering the faller and non-faller groups, fallers did, as a group, display a performance above the 13-s cutoff while non-fallers did not. This may indeed indicate that fallers do have an increased risk of falling when performing translational movements in daily life.

Furthermore, it has previously been reported that patients with peripheral vestibular disorders have difficulties to stand up from a chair, walk, and sit back down again (TUG) (52). Gill-Body et al. (52) reported a mean of 23.33 s for BVP patients to perform the TUG, which is significantly longer than the 8.26 s of the current BVP population. A possible explanation of this discrepancy may be that 59% of their subjects were above the age of 60, which could be an additional influencing factor. Brown et al. (33), on the other hand, indicated that a score of 13.5 s post-rehabilitation was indicative for an increased risk of falling in a BVP population. The current results are well below this cutoff score and are closer to the post-rehabilitation results of 8.80 s reported by Karapolat et al. (53). Based on these results, the TUG does not seem to be sufficiently challenging to determine balance dysfunctions or fall risk in BVP patients. The current protocol also included the TUG with a cognitive and motor dual-task. Both in fallers and non-fallers, the time needed to perform the TUG-cognitive was higher than the single-task TUG. However, performances were still below the 12.08 s reported by Caixeta et al. (54) in an elderly population with chronic vestibular dysfunction and dizziness. The current results are more comparable to results reported in healthy older adults [9.82 ± 2.39 s; (55)] or elderly without a history of falls [9.7 ± 2.3 s; (32)]. Surprisingly, TUG-motor performances tended to be better in fallers. Overall performances on the TUG-motor in BVP patients were better than the times reported in healthy older adults [11.56 ± 2.11 s; (55)] or elderly without a history of falls [9.7 ± 1.6 s; (32)].

The dynamic balance measures discussed above do not challenge the subjects sufficiently, as they do not involve multiple sensory perturbations. Therefore, the Tinetti test and FGA were added to the current protocol, as they contain a combination of more advanced and challenging balance tasks which also approximate the BVP patients' daily activities. The results of the Tinetti test indicate that both fallers and non-fallers are at a medium risk of falling (34). However, the Tinetti test lacks discriminative ability as no differences were found between fallers and non-fallers. Additionally, although the FGA contains several high-level balance tasks, such as walking with horizontal and vertical head movements, walking with eyes closed, walking backwards, or walking with a reduced base of support (35), no differentiation between fallers and non-fallers could be made here as well. The current results on the FGA are comparable to those reported by Marchetti et al. (56) in a vestibular population (19 ± 5.5 points).

The results presented above thus indicate that the performances of fallers on the currently used clinical balance measures are comparable to the performances of non-fallers. Therefore, further investigations on whether clinical balance measures are able to detect fall risk and, if so, are able to distinguish fallers and non-fallers in a BVP population are needed.

### Gait Variables and Fall Risk

Schniepp et al. (11) investigated differences in the spatiotemporal parameters of gait during slow, preferred, and fast walking to determine predictive parameters for falls in BVP patients. For slow walking, significant differences were found for the base of support, where fallers exhibited a wider base of support, and increased coefficients of variation of stride time and length. In the current sample, no significant differences were found between fallers and non-fallers, though it should be noted that the slow walking speeds of the present fallers and non-fallers were significantly higher than the slow walking speeds of the subjects in the study of Schniepp et al. (11) and even were comparable to their preferred walking speeds. Especially during the preferred walking speed condition in the study of Schniepp et al. (11), patients with a history of falls exhibited a slower walking speed combined with a broadened base of support and prolonged double-support phase as compared to non-fallers. These alterations are hypothesized to reflect compensatory strategies to stabilize the impaired walking performance (57), test these differences are completely lacking in the current BVP population, which again could be the result of the higher walking speed of the present sample. It has been shown that, during faster walking and increased cadence, the vestibular influence on the lower limb muscles is selectively suppressed (58, 59) and the direction and variability of walking are less affected by vestibular perturbations (60–62). Therefore, BVP patients may utilize a higher walking speed to suppress the inaccurate vestibular information. For the fast walking speed condition, no differences were found between fallers and non-fallers in both the study of Schniepp et al. (11) as well as in the present study.

Concerning the spatial margins of stability, no differences were found for the AP and ML MoS between fallers and non-fallers during slow, preferred, or fast walking. This can be related to the lack of differences in walking speed for the AP MoS, as the AP MoS is directly dependent on the walking speed (63). However, in a previous study, it was noted that BVP patients primarily use the single- and double-support phases to control the AP MoS instead of walking speed (64), yet again no differences in single and double support were found between fallers and non-fallers in this study. For the ML MoS, step width is the most determining factor for increasing or reducing the MoS, as an increased step width increases the distance between the XCoM and CoP (64–66). Here no significant differences in step width were found between fallers and non-fallers as well, thus resulting in no significant differences in ML MoS.

It has been suggested that including non-preferred slow or fast walking modes could be beneficial in the assessment of BVP patients (11). However, the current results give no indications that the integration of slow or fast walking modes would be of added value.

### Methodological Considerations

Some limitations are to be considered. The current sample of BVP patients is rather small (*n* < 30), introducing a potential selection bias and covering a wide variety of clinical presentations, with half of the sample suffering for more than 10 years from BVP. This may be the cause of the lack of differences found between fallers and non-fallers and could be countered when including more subjects or by including newly diagnosed BVP patients (i.e., not in the chronic phase). An interesting research question for future research could therefore be whether the status of compensation could be used to separate fallers from non-fallers.

Concerning vestibular testing, during rotational chair testing, a frequency of 0.05 Hz was used instead of a frequency of 0.1 Hz as described in the diagnostic criteria of the Barany Society (3). Additionally, while saccular function was determined through c-VEMP testing, information concerning utricular functions is lacking, as no o-VEMP testing was performed. Furthermore, concerning the included population, no control group (e.g., unilateral vestibular hypofunction patients, healthy controls) was included for comparison on the different outcomes.

As the included subjects performed a wide variety of outcome measures, which were not randomized, the results may be subject to potential order effects, e.g., the performances of the subject could be influenced due to the order of the different outcome measures or due to fatigue. However, the subjects were given ample time to rest between each item to reduce the influence of fatigue during testing. As a result, none of the subjects reported to be fatigued during testing or after the testing was completed. In addition, vestibular function testing (vHIT, calorics, and rotatory chair) was performed on a different day than the other outcome measures. On the second day of testing, the subject performed the outcome measures in the following order: PROMs (ABC, DHI)—clinical balance measures [static balance testing, FTSST, TUG with(out) dual tasks, Tinetti test, FGA]—gait variables—dynamic visual acuity testing. All items discussed above may therefore limit the generalizability of the results of the present study.

Concerning the assessment of fall history, fall history was obtained retrospectively and was self-reported, which might be subject to recall bias. We also did not investigate whether the included subjects had any concomitant peripheral (poly)neuropathy, which, as reported by Schniepp et al. (11), might increase the risk of falling even more. Other (chronic) comorbidities present in BVP patients (39), such as but not limited to, cardiovascular disorders, diabetes mellitus, ophthalmological disorders, and otolaryngological disorders may also have an important part in the risk of falling. Relative to these comorbidities, the use of (multiple) medications could also be a determining factor. Therefore, a prospective study countering all the shortcomings above could provide additional imperative information to predict those BVP patients prone to falling.

### Future Directions

In the current study, factors related to vestibular functioning, balance performance, and gait performance were unable to distinguish fallers from non-fallers in a bilateral vestibulopathy population. These physical measurements seem less able to do so compared to psychological factors, such as balance confidence (ABC) or self-perceived handicap (DHI). Fallers, in general, showed better results on vestibular function tests and performed on the same level as non-fallers with regards to balance and gait outcome measures. The results on PROMs, on the other hand, indicated that fallers were more likely to report a more severe impairment or lower level of functioning. These results may indicate that a patient's beliefs concerning their capabilities are more important than the moderately or severely affected physical performance when distinguishing fallers from non-fallers, as patients may have a better understanding of their capabilities than what is indicated by physical tests (67). Furthermore, when a person's balance confidence and self-efficacy are reduced, they will be more likely to alter their behavior and avoid those situations and activities which they believe will cause a fall (67, 68). Therefore, it should be kept in mind, for future studies, that the performance on physical measures does not necessarily correspond with the perception of BVP patients. Thus, outcome measures addressing the self-efficacy and fear of falling should be incorporated in future research to investigate whether these are able to distinguish fallers from non-fallers. As has been presented in the current study and the study of Dobbels et al. (10), the DHI may be a useful tool. In addition, information regarding physical activity could provide valuable insights on contextual information influencing behavior and falls. Therefore, below are other suggested PROMs which could be of aid in future research and assessment protocols.

#### Fear of Falling Avoidance Behavior Questionnaire

The Fear of Falling Avoidance Behavior Questionnaire (FFABQ) (69) is a self-reported assessment which quantifies the individual's avoidance of specific activities due to fear of falling. The FFABQ contains 14 items stated as “Due to my fear of falling, I avoid … (activity or participation),” scored using a five-point ordinal scale (0 = completely disagree to 4 = completely agree), resulting in a total possible score of 84 points. A higher score indicates a greater limitation of activity and restriction of participation as a result of fear of falling.

#### Ambulatory Assessment of Physical Activity

The term “ambulatory assessment” is an umbrella term covering a wide range of methods with the aim of studying an individual's real-life processes and gathering data on behavioral, biological, and physiological levels (70, 71). This way, several challenges related to traditional methods relying on retrospective self-reports (e.g., interviews and questionnaires) are bypassed, and consequences of physical activity as they unfold in daily life and in the individuals' natural setting can be uncovered (71). “Ambulatory assessment” thus does not only cover ambulatory movement assessments (i.e., walking), but it also contains an assessment of physiological and environmental parameters using sensors (e.g., accelerometers, geolocation tracking), paper-and-pen diaries, or electronic diaries. This way, retrospective biases can be minimized as data can be assessed near real time. We refer to Reichert et al. (71) for a comprehensive overview.

Additionally, as a recent review concerning balance performance in bilateral vestibulopathy has reported, information concerning locomotion-incorporating activities from daily life is still lacking (13). The current study took a first step in mitigating this lacuna by including clinical balance measures, such as the TUG with or without dual tasks, Tinetti test, and FGA. Additionally, it has been suggested that BVP patients perform poorly on movement strategies (e.g., reactive balance control), control of dynamics (e.g., gait), orientation in space (e.g., perception of verticality), and cognitive processing (e.g., attention) (13). The balance measures described below could potentially be implemented in the assessment protocols for BVP patients and could potentially aid in differentiating fallers from non-fallers.

#### BESTest

Many clinical balance measures are designed in such a way that only a single balance system is tested. However, balance control is very complex and thus involves many different underlying systems (72–74). Therefore, Horak et al. (75) developed a clinical balance measure based on the concept that postural control results from a set of interacting systems: the BESTest. The BESTest contains a total of 36 items, subdivided over six sections. Each item is scored on a four-level ordinal scale from 0 (worst performance) to 3 (best performance). The scores for each section, as well as the total score, are provided as a percentage of the total points. The different sections include (1) biomechanical constraints, (2) stability limits/verticality, (3) anticipatory postural adjustments, (4) postural responses, (5) sensory orientation, and (6) stability in gait. Some of the balance tasks included in the BESTest have been borrowed from other clinical tests, such as the Functional Reach Test, Fregly Single-Limb Stance Test, Berg Balance Scale, Clinical Test of Sensory Interaction on Balance, Dynamic Gait Index, and Timed Up and Go. By incorporating these different sections and items, the BESTest assesses all aspects of balance control on which BVP patients might experience difficulties. A downside of the BESTest, however, is the time needed to complete it, as it can take up to 30 min.

Therefore, the mini-BESTest (76) was developed. As it consists of 14 items from the original BESTest (75), it can be conducted in 10 to 15 min as opposed to 20–30 min.

#### Community Balance and Mobility Scale

The Community Balance and Mobility (CB&M) scale evaluates high-level deficits in gait, balance, and mobility and was originally developed for high-functioning young and middle-aged adults with traumatic brain injuries (77). The CB&M consists of 19 tasks representing underlying motor skills necessary for functioning in the community. It includes different aspects of posture and movement, such as multitasking, sequencing of movement components, and complex motor skills. The multitasking (i.e., cognitive processing) aspects assess the control of posture while performing multiple tasks at the same time, such as walking while maintaining gaze on a target. Sequencing of movement components (i.e., movement strategies) includes tasks, such as walking and picking up an object from the floor. Lastly, components related to complex motor skills (i.e., control of dynamics) included crossing one foot over the other while moving laterally.

#### Cognitive–Motor Dual-Task Assessment

The recently published 2BALANCE protocol (78) consists of static and dynamic postural tasks, combined with different cognitive tasks, and therefore mitigates the lacuna concerning cognitive processing. The cognitive tasks assess the different cognitive domains identified to be impaired in persons with vestibular disorders: visuospatial cognition, processing speed, working memory, and response inhibition. Examining these different tasks may provide the opportunity to elaborate on the impact of vestibular dysfunction on cognitive and motor task performances in single- and dual-task settings, resembling everyday situations.

## Conclusion

Based on the data presented in the current study, none of the included outcome measures, at this time, seem useful in distinguishing BVP patients with a history of falls from those who did not fall. However, the results of this study may pave the way toward determining useful outcome measures predicting the risk of falling in bilateral vestibulopathy. The vestibular function of fallers tended to be better than that of non-fallers, while non-fallers, in general, showed the same fall risk as non-fallers on the clinical balance measures. The biomechanical balance measures also did not show any differences between fallers and non-fallers. On the other hand, fallers did tend to report a worse self-perceived disability. These results emphasize the need for a more thorough investigation concerning the predictive ability of outcome measures for BVP patients' fall status. The work-up of BVP patients should therefore consist of outcome measures covering all levels of the International Classification of Functioning, Disability, and Health model. Additionally, it is proposed to include PROMs related to fear of falling and balance measures that are as closely related to activities of daily life.

## Data Availability Statement

The datasets presented in this study can be found in online repositories. The names of the repository/repositories and accession number(s) can be found at: https://doi.org/10.6084/m9.figshare.13019519.

## Ethics Statement

The studies involving human participants were reviewed and approved by Ethics Committee of the University of Antwerp/Antwerp University Hospital. The patients/participants provided their written informed consent to participate in this study.

## Author Contributions

NH conceived and designed the study, acquired, analyzed, and interpreted the data, and wrote the manuscript with input from all authors. BD and JM acquired and stored data and critically revised the manuscript for important intellectual content. RV interpreted the data and critically revised the manuscript for important intellectual content. WS, AH, LV, and VV conceived and designed the study and critically revised the manuscript for important intellectual content. All authors contributed to the article and approved the submitted version.

## Conflict of Interest

The authors declare that the research was conducted in the absence of any commercial or financial relationships that could be construed as a potential conflict of interest.
