# Supplementary material for: Paving the Way Toward Distinguishing Fallers From Non-fallers in Bilateral Vestibulopathy: A Wide Pilot Observation
Source: Front Neurol. 2021 Mar 1;12:611648. doi: 10.3389/fneur.2021.611648 (PMC7957054; doi:10.3389/fneur.2021.611648)
Supplement: Supplementary file 1 [file Table_1.DOCX]

Supplementary Material

# Biomechanical Balance Measures

Of 23 of the included BV-patients an instrumented 3-dimensional gait analysis was performed at a movement analysis laboratory equipped with 8 infrared cameras (Vicon T10, 100 Hz., ©Vicon Motion Systems Ltd., Oxford, UK, 100 fps, resolution 1 Megapixel (1120×896), 3 AMTI type OR 6–7 force plates (1000 fps, 46×50×8 cm) and 1 AccuGait® (1000 fps) force plate. Reflective markers were placed on anatomical landmarks on the subject’s body corresponding to the left and right first metatarsal (toe), lateral malleolus (ankle), major trochanter, the sacrum and C7.
Subjects walked barefoot over a 12-meter-long walkway at self-selected slow, preferred and fast walking speed. Marker trajectories were labelled using the Vicon Nexus 1.x.x software, based on ankle marker trajectories and force plate date the event of foot strike and foot off were determined. Gait cycles were calculated based on left and right heel marker trajectories.
The .c3d-files were exported to Matlab (R2019a for Windows) and through a custom-written script the spatiotemporal parameters of gait and the spatial margins of stability were calculated.

## Spatial Margins of Stability

For The extrapolated centre of mass (XCoM) is defined as the vector sum of the centre of mass position and a proportion of its velocity as described by Hof, et al. [1]. The XCoM used in this study was adapted to the reduced kinematic model [2] used for the BV-patients, as described above. The current method differs from that of Hof, et al. [1], as in the current study the position of the centre of mass (CoM) was estimated by using the average of the markers placed on the major trochanter for the reduced model (BV-patients) or Anterior Superior Iliac Spine for the Plug-In-Gait model (HCs).

$$XCoM= \frac{P_{m1}+ P_{m2}}{2}+ \frac{0.5(\frac{v_{m1}+ v_{m2}}{2}+v_{C7})}{\sqrt{\frac{g}{l}}}$$

With $P_{m1}$, $P_{m2}$ corresponding to the positions of the left and right major trochanter marker positions in the reduced model and the left and right Anterior Superior Iliac Spine marker positions in the Plug-In-Gait model, representing the vertical projection of the CoM. $v_{m1}$, $v_{m2}$, and $v_{C7}$ are the velocities of the trochanter/Anterior Superior Iliac Spine and C7 markers respectively, representing the velocity of the CoM. Lastly, $g$ represents the acceleration of gravity (9.81 ms^-2^) and $l$ is defined as the leg length defined as a fraction of body height: $0.530*Body height (mm)$[3].

## Spatial margins of stability

The MoS were calculated based on the equations defined by Hof, et al. [1]. The medio-lateral MoS was defined in this study as the minimum distance between the boundary of the BoS, i.e. the ankle marker ($P_{Ankle}$), and the XCoM along the medio-lateral axis during the single support phases. The medio-lateral axis was defined as the axis in the transverse plane, perpendicular to the walking direction derived from the CoM coordinates.

$$ML MoS= P_{Ankle}-XCoM$$

The anterior-posterior MoS was defined in this study as the distance between the boundary of the BoS of the leading foot, i.e. the ankle marker ($P_{Ankle}$), and the XCoM along the anterior-posterior axis at foot touchdown. With the anterior-posterior axis being defined as the axis in the transversal plane, parallel to the walking direction derived from the CoM coordinates. Although it should be noted that, by using the ankle marker of the leading foot to define the posterior border of the base of support, a slight but systematic underestimation of the AP MoS has been made [4].

$$AP MoS=P_{Ankle}- XCoM$$

1. Hof AL, Gazendam MG, Sinke WE. The condition for dynamic stability. *J Biomech.* 2005;38(1):1-8, 10.1016/j.jbiomech.2004.03.025

2. Süptitz F, Catalá MM, Brüggemann G-P, Karamanidis K. Dynamic stability control during perturbed walking can be assessed by a reduced kinematic model across the adult female lifespan. *Human Movement Science.* 2013;32(6):1404-1414, <https://doi.org/10.1016/j.humov.2013.07.008>

3. Winter DA. *Biomechanics and Motor Control of Human Movement.* Wiley; 2009.

4. Hak L, van Dieen JH, van der Wurff P, Houdijk H. Stepping asymmetry among individuals with unilateral transtibial limb loss might be functional in terms of gait stability. *Phys Ther.* 2014;94(10):1480-1488, 10.2522/ptj.20130431
